# Measuring Humoral Immune Responses to SARS-CoV-2: A Comprehensive Review of Serological Assays

**DOI:** 10.3390/vaccines14050395

**Published:** 2026-04-28

**Authors:** Huijing Xue, Katarzyna Haynesworth, Heidi A. Hempel, Troy J. Kemp, Ligia A. Pinto

**Affiliations:** Vaccine, Immunity and Cancer Directorate, Frederick National Laboratory for Cancer Research, Frederick, MD 21701, USA; huijing.xue@nih.gov (H.X.); kemptj@mail.nih.gov (T.J.K.)

**Keywords:** COVID-19, serological assays, assay standardization, vaccine immunogenicity

## Abstract

The COVID-19 pandemic highlighted the critical role of serological assays in understanding antiviral immune responses, monitoring vaccine efficacy, and informing public health strategies. This review provides a comprehensive overview of commonly used SARS-CoV-2 antibody detection methods, focusing on binding and neutralization assays. Antibody binding assays, including enzyme-linked immunosorbent assays (ELISAs), chemiluminescence immunoassays (CLIAs), lateral flow immunoassays (LFAs), and multiplex platforms, enable the rapid and high-throughput detection of immunoglobulin isotypes against various viral antigens. Neutralization assays, including live-virus, pseudovirus (PsV), and surrogate assays, offer functional insights into the ability of antibodies to prevent viral entry, though they often require higher biosafety levels and optimization. Serological assays, primarily antibody binding assays and several surrogate neutralization assays, received Emergency Use Authorization (EUA) during the pandemic, supporting seroprevalence efforts. Antibody binding assays and neutralization assays were also widely used in vaccine immunogenicity studies. Despite many standardization initiatives, assay standardization and data harmonization remain challenging and require further efforts. The choice of assay should be guided by study goals: antibody binding assays are preferred for high-throughput monitoring and epidemiological studies, while neutralization assays are essential for assessing functional immunity and variant-specific neutralization and protection.

## 1. Introduction

Coronavirus Disease 2019 (COVID-19), caused by Severe Acute Respiratory Syndrome Coronavirus 2 (SARS-CoV-2), rapidly spread since its first recognized emergence in December 2019. Due to the emerging variants, COVID-19 has posed a threat to public health and the global economy. According to the World Health Organization (WHO), the cumulative number of confirmed cases as of 22 October 2025 was >778.7 million, with >7 million deaths attributed to COVID-19 [1]. SARS-CoV-2 is highly contagious and primarily affects the respiratory system, although long-term effects involving multiple organ systems are well documented [2]. Following infection, SARS-CoV-2 replicates in respiratory epithelial cells and can trigger dysregulated immune responses, including excessive inflammation and cytokine release, which can contribute to disease severity [3]. Clinically, COVID-19 ranges from asymptomatic or mild disease to severe pneumonia, acute respiratory distress syndrome (ARDS), thrombotic complications, and multi-organ dysfunction, with some individuals experiencing persistent post-acute sequelae (“long COVID”) [3,4].

As a membrane-enveloped virus, SARS-CoV-2 has four structural proteins: spike (S), membrane (M), envelope (E), and nucleocapsid (N) [5]. The spike protein (S1 and S2 subunits) facilitates the virus entry into host cells by binding to the Angiotensin-Converting Enzyme 2 (ACE2) receptor through the receptor-binding domain (RBD) within its S1 subunit [5]. The nucleocapsid protein is vital for virus replication and transcription [5]. Antibodies targeting different viral proteins provide complementary information: anti-spike (S) antibodies are directly relevant for virus neutralization, vaccine-induced protection, and immune recognition of emerging variants, making them critical immunity markers. In contrast, anti-nucleocapsid (N) antibodies primarily indicate prior natural infection, since most currently licensed vaccines are spike-based and thus do not elicit N-specific responses. Antibodies (Abs) against viral proteins can be measured using antibody binding assays, such as enzyme-linked immunosorbent assays (ELISAs) and multiplex assays [6,7,8]. Furthermore, binding antibodies can be categorized into neutralizing (NAbs) and non-neutralizing (nNAbs) antibodies. NAbs are considered important mediators of effective humoral immunity against SARS-CoV-2, as they can prevent infection by blocking the virus’s entry into host cells [5], and NAbs can be assessed through neutralization assays [9,10]. While several reviews have examined individual serological assay platforms, a comprehensive framework integrating antibody binding and neutralization assays, together with considerations of standardization, harmonization, and practical guidance for assay selection in research and clinical contexts, remains limited. In this review, we provide a comprehensive overview of current SARS-CoV-2 binding and neutralization assays, discussing their methodological approaches, strengths, limitations, and applications in the context of infection, vaccination, and emerging viral variants.

## 2. Measuring Total Binding Antibody Levels

### 2.1. Singleplex Assays

#### 2.1.1. Enzyme-Linked Immunosorbent Assays (ELISAs)

ELISAs for measuring anti-SARS-CoV-2 antibodies typically utilize an indirect assay format. This involves passively absorbing the antigen onto a solid surface, usually a 96-well polystyrene plate, and then adding antibodies from clinical samples, such as serum. Typically, ELISAs use full-length spike, receptor-binding domain (RBD), or nucleocapsid proteins as coating antigens, depending on the assay design and the desired specificity for infection or vaccination-induced antibodies [11]. Subsequently, secondary anti-species antibodies conjugated to enzymes, such as horseradish peroxidase (HRP), catalyze colorimetric reactions (e.g., with tetramethylbenzidine, TMB) to provide a measurable signal proportional to antibody binding, which is assessed using a spectrophotometer. This system allows flexibility in quantifying specific immunoglobulins (IgG, IgA, IgM). Several studies detail the development and validation of serology ELISA platforms, including antigen coating options, calibration to the World Health Organization International Standard (WHO IS) for anti-SARS-CoV-2 immunoglobulin [6,11,12,13,14,15], and the U.S. Food and Drug Administration (FDA) has granted Emergency Use Authorization (EUA) for multiple assays (Table 1) [16].

The initial COVID-19 antibody assays developed during the pandemic focused on the spike antigen following the publication of its sequence [6]. Some of the initial ELISAs utilized a two-step process: screening for antibodies against RBD, which is relatively easy to produce, and confirming positive cases with the full-length spike, which is challenging to express due to its large size, complex trimeric structure, and extensive glycosylation patterns [6]. Recombinant spike or RBD proteins are commonly expressed in mammalian or insect cell systems to preserve structural epitopes critical for serological assays. The two-step strategy enabled high-throughput, large-scale serosurveys [6]. Additionally, ELISAs can be performed on a 384-well format to increase throughput [14]. To facilitate comparability across studies and platforms, antibody levels are increasingly reported in standardized units, such as binding antibody units per milliliter (BAU/mL), aligned with the World Health Organization international standard (WHO IS) [25]. Several ELISA-based serological assays have received FDA EUA, with representative examples summarized in Table 1, showing strong performance characteristics in clinical validation studies [16]. These assays typically target spike, RBD, or nucleocapsid antigens and are designed for quantitative or semi-quantitative detection of SARS-CoV-2-specific antibodies in clinical samples.

Subsequent developments in ELISA platforms focused on targeting specific immunoglobulins, nucleocapsid proteins, and emerging viral variants. Comparative analyses of antibody isotypes have shown that IgA, IgM, and IgG responses exhibit various temporal patterns and diagnostic utilities. While IgM and IgA are typically considered early antibody responses that wane more rapidly and can indicate recent infections, SARS-CoV-2 infection could elicit overlapping or near-concurrent IgM and IgG responses, with IgG persisting longer and serving as a marker of prior exposure or vaccination [12,13,26,27]. Assay performance varies across assays, protocols, and laboratories, due to differences in Standard Operating Procedure (SOP) implementation, antigen quality, and additional technical factors. Spike-based ELISAs may provide higher specificity [13], while nucleocapsid-based assays can be more sensitive for early infection [28]. Longitudinal studies indicate that anti-RBD IgG can remain detectable for up to a year, while anti-nucleocapsid IgG levels decline more rapidly [12]. As anti-nucleocapsid antibodies are induced primarily by natural infection, they can help differentiate between vaccinated and infected individuals, although assay sensitivity can decline over time and interpretation should account for vaccine platform and waning immunity [29,30]. Given that emerging viral variants, including Omicron sublineages, can partially evade neutralizing antibody recognition [19], continuous optimization of ELISA designs and antigen selection remains important to ensure assay sensitivity and clinical relevance, particularly for assays targeting mutation-prone regions such as RBD. Assay optimization strategies for emerging SARS-CoV-2 variants include incorporating variant-specific spike or RBD sequences, using multivalent antigen panels, and designing epitope-focused assays to distinguish between antibody responses against different variants.

#### 2.1.2. Chemiluminescence Immunoassay (CLIAs)

Chemiluminescence immunoassays (CLIAs) share the same antigen-antibody binding principle as colorimetric ELISAs but differ in their signal detection mechanism. In CLIAs, antigens are often conjugated to magnetic particles, and patient samples are incubated to allow binding of antibodies to viral antigens. A secondary antibody labeled with a chemiluminescent enzyme or tag then generates light upon substrate addition, and the emitted signal correlates with antibody concentration. These platforms can be automated, offering improved sensitivity, throughput, signal-to-noise ratio, reagent stability, and ease of use [31,32]. While both CLIA and ELISA achieve high specificity for SARS-CoV-2 antibodies, reported sensitivities vary across studies, with some showing higher sensitivity for CLIA and others for ELISA [33,34,35]. The performance of different platforms could be driven by assay design, antigen selection, assay cutoffs, and sampling time, rather than the label chemistry alone [34,36].

Several CLIA-based serological assays have received FDA EUA, including high-throughput platforms targeting spike, RBD, or nucleocapsid antigens, with the capability to detect IgG, IgM, or total antibodies in clinical samples with accuracy. These FDA-authorized CLIA assays have demonstrated high sensitivity and specificity [16]. In addition to FDA-authorized platforms, other commercially available CLIA systems have been developed globally using similar detection principles. These platforms commonly utilize RBD-based antigens and have demonstrated high sensitivity, reproducibility, and suitability for automated clinical workflows [20,37]. Overall, CLIA platforms leverage chemiluminescent detection to achieve high sensitivity, reproducibility, and throughput, making them well suited for large-scale serological screening and monitoring of vaccine-induced immune responses.

#### 2.1.3. Lateral Flow Assay (LFA)

Lateral flow immunoassays (LFAs) are based on the same antigen-antibody binding principle as ELISA and CLIA but use a simple immunochromatographic strip format designed for rapid, point-of-care use. Antibodies in samples bind to labeled viral antigens on nitrocellulose strips and form visible lines through detection particles such as colloidal gold or fluorescent probes. Band intensity correlates with antibody concentration, enabling qualitative or semi-quantitative interpretation [38,39]. Among serological assays for SARS-CoV-2, LFAs provide faster results, minimal instrumentation, and easy field deployment, but typically lower analytical sensitivity and greater operator subjectivity, particularly when interpreting visually weak bands [34,40]. Their simplicity, portability, and low cost make them suitable for rapid screening and field surveillance. These point-of-care tests typically detect IgM and IgG antibodies against spike or nucleocapsid antigens and have demonstrated good diagnostic performance, with reported sensitivity and specificity generally exceeding 90%, especially when IgM and IgG results are interpreted in combination [16]. Further developments have expanded the application of LFAs beyond single-analyte detection. Several multiplex LFAs have been developed to simultaneously detect distinct SARS-CoV-2 immunoglobulin isotypes or antigens from multiple respiratory pathogens using a single swab sample, thereby enhancing diagnostic efficiency and epidemiological surveillance [21]. Recent advances have also adapted the LFA platform to assess neutralizing activity [41]. However, in general, they are less optimal for applications requiring high sensitivity or quantitative precision, such as vaccine immunogenicity studies. Careful verification of band reading, training, and sample type remains essential to ensure reliability. Additional developments in this area are still warranted to expand the applicability of LFA.

Multiple studies employing ELISA, CLIA, and LFA platforms have demonstrated good correlations between spike-specific binding antibody titers and neutralizing activity [42,43,44]. These findings support the potential use of such assays as practical surrogates for neutralization testing, allowing effective monitoring of humoral immune responses following infection or vaccination. They are also valuable for assessing vaccine-induced immunity across populations and for identifying suitable donors for convalescent plasma therapy. Several of these serological assays have received EUA from the FDA for clinical or surveillance applications [17] (Table 1). These assays target spike, RBD, or nucleocapsid antigens and detect various immunoglobulin classes, including IgG, IgM, IgA, or total antibodies. Several commercial serological assays have undergone iterative updates, including improved antigen design and the adoption of multiplexed assays. In parallel, assay outputs transitioned from qualitative or semi-quantitative formats toward more standardized and quantitative measurements, with increasing alignment to the WHO IS and reporting in BAU/mL to enhance inter-laboratory comparability [45,46]. Additional updates include improvements in antigen design, automation, and throughput, as well as the development of surrogate neutralization assays that enable functional assessment without live virus [10]. Collectively, these assays provide validated options for monitoring vaccine responses, evaluating prior infection, and supporting public health studies.

### 2.2. Multiplex Assays

Multiplex immunoassays enable the detection of multiple analytes in a small sample volume in a single well. This offers numerous advantages, including maximizing the information obtained from a single sample, reducing sample volume requirements and labor. Additionally, multiplex assays allow for the concurrent testing of antibodies against other coronaviruses and other pathogens, enabling cross-reactivity and cross-protection evaluations. Common multiplex platforms include bead-based immunoassays such as the Luminex platform, as well as microtiter plate-based arrays (Table 1).

#### 2.2.1. Bead-Based Immunoassays

A bead-based assay platform, such as the Luminex system, utilizes magnetic microspheres made from carboxylated polystyrene polymers that enable proteins to be attached to the bead surface [47]. Each bead set is dyed to a distinct spectral region, enabling unique identification of up to 500 analytes [47]. Various bead-antigen couplings are incubated with serum or plasma to capture the corresponding antibodies. Subsequently, a secondary anti-species antibody conjugated to the fluorescent reporter molecule binds to enhance detection. The Luminex reader functions as a dual-laser cytometer, analyzing suspended beads, capturing multiple readings for each bead set, and reporting the median fluorescence intensity (MFI) for each analyte [47]. Bead-based protocols provide an improved antigen-displaying surface compared to ELISA [25], and the bead-based assay can achieve a broader dynamic range of 4.5-5.5 logs (depending on the reporter channel) [48]. However, the effectiveness of MFI-based methods can be limited due to signal saturation. At high analyte concentrations, MFI values can plateau, leading to reduced assay sensitivity and potential inaccuracies in quantification [25]. To mitigate this, optimal sample dilution and the use of a universal standard such as the WHO IS are recommended.

Several studies have suggested that some Luminex assays correlate well with commercial ELISA/CLIA tests in detecting antibodies against SARS-CoV-2 proteins [49], meanwhile offering advantages such as higher throughput and high sensitivities [50]. The Luminex-based multiplex platform can be used to screen various SARS-CoV-2 antigen sources and identify optimal antigen combinations to improve assay performance. A study screened over 100 antigens and demonstrated that combining two of the three key antigens, including the spike trimer, the S1 domain of the spike, and the C-terminal domain of the nucleocapsid, achieved high sensitivity (99.7%) and specificity (99.4%), highlighting the advantage of multi-antigen approaches in serological evaluation [7]. While multiplex immunoassays provide high-throughput, multidimensional profiling of antibody responses, they require specialized instrumentation, are more complex to implement, and incur potentially higher costs, limiting their routine use in clinical laboratories. In addition, increasing the number of analytes may introduce antibody-antibody interference and non-specific interactions, which can affect assay specificity and signal accuracy. As a result, careful assay optimization and validation are required when expanding multiplex panels.

#### 2.2.2. Microtiter Plate-Based Arrays

Microtiter plate-based arrays use a planar format in which multiple antigens are immobilized as discrete spots within each well on a standard 96- or 384-well plate, allowing simultaneous detection of multiple analytes within the same sample. Serum or plasma antibodies bind to specific antigens, and labeled secondary antibodies generate colorimetric, fluorescent, or chemiluminescent signals. Spot-specific intensities are quantified, allowing multiplexed readouts per well. The number of spots per well varies, depending on spot size, well area, the resolution of printing, and detection. For example, Meso Scale Discovery (MSD) panels employ 10-spot, 96-well plates coated with different antigens on each spot [51]. Researchers developed a 96-well multiplex platform with automated imaging capable of accommodating 59 spots per well while maintaining high sensitivity (97.8%) and specificity (97.7%) [52]. Microtiter plate-based arrays provide a flexible antigen layout; however, plate-based arrays may be more susceptible to spot-to-spot variability and crosstalk between neighboring spots, often requiring careful optimization of sample dilution and imaging conditions [52,53].

Several plate-based multiplex platforms have been adapted for SARS-CoV-2 serological applications. For example, commercial electrochemiluminescence panels have been developed to simultaneously quantify antibodies against multiple viral antigens, including those from SARS-CoV-2 and its variants, as well as SARS-CoV-1, MERS-CoV, and other respiratory infections [24]. The multiplex serology panels have been widely implemented in vaccine evaluation and immunogenicity studies, including large-scale phase 3 clinical trials of mRNA and adenoviral vector vaccines [54,55]. Beyond high-throughput detection, multiplex datasets enable detailed immune profiling, supporting the identification of antibody signatures associated with protection or disease severity. In addition, integration with advanced analytical approaches, such as multivariate modeling, may further leverage and enhance the interpretability and translational value of these data.

In addition to the assays summarized above, peptide-based membrane immunoassays, in which overlapping SARS-CoV-2-derived peptides are immobilized on nitrocellulose membranes, have been used to characterize antibody binding to linear viral epitopes [56]. However, these approaches are primarily applied in epitope mapping studies and are less commonly used in validated or standardized serological assays for quantitative antibody measurement.

#### 2.2.3. Studies Utilizing Multiplex Immunoassays

Multiplex immunoassays enable assessing different immunoglobulin isotypes and viral proteins simultaneously, providing valuable insights into disease progression, vaccination status, and the durability of the immune response. Researchers developed a Luminex-based multiplex immunoassay to detect IgA, IgG, and IgM against nucleocapsid, Spike S1, S1-RBD, and S1-NTD, which could distinguish severe/critical from mild/moderate infections and differentiate between natural infections and vaccinations [57]. Longitudinal analyses using a Luminex-based multiplex assay showed that spike and RBD antibodies had similar half-lives across IgM, IgA, and IgG, while spike-specific IgM decayed faster than IgA and IgG, and anti-nucleocapsid IgG declined most rapidly [26].

Emerging evidence also indicates that IgG subclass distribution can shift over time with repeated antigen exposure, particularly following mRNA booster vaccination. While early responses to SARS-CoV-2 infection or primary vaccination are typically dominated by IgG1 and IgG3, subsequent antigen exposure has been associated with increased proportions of IgG2 and, notably, IgG4 antibodies [58]. Multiplex platforms have facilitated the characterization of IgG subclass responses, providing further resolution of antibody quality. A study indicated a potential association between the IgG4 switch following mRNA booster vaccination and a higher risk of breakthrough infection [59]. Another study suggested that, while elevated IgG4 could reduce antibody-dependent cellular cytotoxicity (ADCC) responses, it may enhance FcγRI- and FcγRIIa-mediated functions such as antibody-dependent cellular phagocytosis (ADCP) under conditions of lower overall antibody titers, particularly against SARS-CoV-2 variants [60]. Multiplex subclass profiling has also revealed differences in subclass distribution across disease severity and vaccine platforms, highlighting its utility in further dissecting qualitative aspects of humoral immunity beyond total antibody levels [61,62].

Due to the high mutation rate of SARS-CoV-2, it is beneficial to utilize multiplex technologies to evaluate immune response against several viral variants simultaneously. Studies evaluated antibodies against nine SARS-CoV-2 antigens, including nucleocapsid and spike proteins from the Wuhan, Alpha, Beta, and Gamma variants, and identified distinct serological signatures reflecting variant-specific immune recognition [8]. Another study examined the antibody responses to 15 SARS-CoV-2 variants in patients with non-small-cell lung cancer (NSCLC) and healthy donors using microtiter plate-based panels [63]. They observed reduced spike-specific IgG levels for Delta and Beta variants relative to the ancestral strain.

Multiplex assays can also profile antibodies against related coronaviruses and other respiratory pathogens. A study utilizing multiplex plates suggested that IgG, IgM, and IgA antibodies against alphacoronaviruses (HCoV-229E, HCoV-NL63) and betacoronaviruses (HCoV-HKU1, HCoV-OC43) remained stable for over 200 days, and that SARS-CoV-2 infection can enhance cross-reactive immunity against other betacoronaviruses, including SARS-CoV-1 [26]. Various commercial multiplex panels are also available, targeting diverse SARS-CoV-2 antigens, variants, and additional respiratory pathogens [22,23]. Overall, multiplex platforms provide a reliable and scalable approach for profiling complex antibody responses, with performance generally comparable to standard ELISA when properly validated.

Collectively, ELISA- and CLIA-based assays are well suited for quantitative and high-throughput applications, particularly in large-scale serosurveillance and vaccine studies, whereas lateral flow assays offer rapid, point-of-care testing often associated with reduced sensitivity and limited quantitative capability. Multiplex platforms provide expanded analytical depth, enabling simultaneous assessment of multiple antigens or antibody classes, and are particularly valuable in research and immune profiling contexts.

## 3. Measuring Neutralizing Antibody Levels

Neutralization assays can be broadly categorized into three types: live-virus, pseudovirus (PsV), and surrogate virus assays. These assays (Table 2) measure the ability of patients’ serum or monoclonal antibodies to inhibit viral infection by 50% or 80% compared to control samples. Each assay requires extensive optimization, such as titrating virus input and selecting appropriate target cells, to achieve reliable, reproducible, and quantitatively comparable results across different batches and studies.

### 3.1. Live-Virus Neutralization Tests

Considered as the gold standard for quantifying neutralization ability against viruses, the live-virus neutralization test directly measures the level of NAbs required to block authentic viral infections [72]. Protocol steps involve incubating live viruses with serum or monoclonal antibodies, followed by infection of susceptible host cells. Classical live-virus neutralization assays often quantify neutralizing activity by measuring inhibition of virus-induced cytopathic effect (CPE) in these cells, providing a functional assessment of protective antibodies [73]. These CPE-based assays are typically qualitative or semi-quantitative. To achieve greater precision, live-virus neutralization assays are commonly performed as the plaque reduction neutralization test (PRNT), the focus reduction neutralization test (FRNT), and the microneutralization test (MNT).

PRNT quantifies neutralization potency by counting plaque reductions caused by viral cytopathic effects, though results can vary due to operator subjectivity [64,74]. FRNT uses immunostaining against viral proteins to visualize the ‘foci’ of infected cells, which plate analyzers can count [75]. Typically, the foci form before plaque formation, leading to a shorter incubation time than PRNT. Traditional PRNT and FRNT are usually performed in 6-well or 24-well plates with limited throughput. MNT refers explicitly to the neutralization tests performed on microplates (allowing medium throughput) but includes various readouts such as cytopathic effect inhibition, immunostaining, or RT-PCR quantification, depending on viral characteristics [67,73]. FRNT can be adapted to 96-well formats, and MNT can utilize immunostaining to visualize viral “foci” [75,76]. Thus, the definitions of FRNT and MNT are sometimes mixed.

The use of live viruses and cells introduces several experimental variables, such as virus-receptor expression level; cell type, density, and morphology; virus infectiousness; and cell or virus chemistry modulation, contributing to significant intra- and inter-assay variation. Precision acceptance for immunological assays generally requires a coefficient of variation (CV) of less than 20%; however, a 25–30% CV is often considered acceptable for cell-based neutralization assays [77]. In addition, live-virus neutralization tests are time-consuming and require both skill and resources to utilize authentic SARS-CoV-2 in a biosafety level 3 (BSL-3) laboratory, limiting their clinical utility for large-scale screening and vaccine evaluation. Optimizing each variable is crucial for enhancing assay repeatability and establishing a robust platform for NAb assessment.

Despite these challenges, live-virus neutralization tests have been used in clinical trials to evaluate vaccine efficacy. A phase II/III trial (COV002) assessed the ChAdOx1 nCoV-19 vaccine using a microneutralization test [78]. In a phase I/II study (NCT04368728), a modified assay was employed to assess the efficacy of the BNT162b1 vaccine, where the omicron spike proteins were further engineered into an mNeonGreen system to evaluate the neutralizing response to three doses of BNT162b2 [79,80]. Increasingly, clinical trials are adopting pseudovirus assays or combining both methods. However, cross-cohort comparison is a huge challenge due to assay variability and inconsistent units. Standardization, optimization, and alignment with WHO IS are essential for improving reliability and comparability across studies.

#### Optimizing Live-Virus Neutralization Tests

Different result readouts have been adapted to reduce reading errors and variability. An optimized fluorescence-based assay using a recombinant SARS-CoV-2 with an mNeonGreen gene insertion was developed, showing a high correlation with PRNT (R^2^ = 0.8512). The fluorescent reporter system decreases incubation time and labor and enables accurate quantification via automated cell imaging of fluorescent, virally infected foci [65]. Similarly, another study introduced a nanoluciferase gene into wild-type SARS-CoV-2, enabling rapid, high-throughput readouts with high sensitivity and strong PRNT correlation (R^2^ = 0.8380) [66]. Quantitative RT-PCR (qRT-PCR) methods directly measure viral RNA in lysed cells, show a strong correlation with conventional assays (R^2^ = 0.8910), and can be operated in a BSL-2 lab since the virus is inactivated during cell lysis [67]. Other variables in need of optimization include virus incubation time, cell density, virus concentration, and normalization models, such as those performed in a study utilizing a microneutralization assay with Vero E6 cells [81]. Similar optimization should be performed in different experimental settings and additional factors like cell types should be included.

While the live-virus neutralization assay is complicated and requires a BSL-3 setting, it represents the most sensitive standard and should serve as a reference method to validate more accessible and reproducible assays.

### 3.2. Pseudovirus Neutralization Test

Instead of using authentic viruses, pseudovirus neutralization tests (pVNT) utilize synthetic replication-defective pseudovirus (PsV), which typically incorporates the surface protein of the original virus to simulate the process of virus-receptor binding and viral entry [82]. These changes allow testing in BSL-2 laboratories, increasing accessibility. PsVs often carry reporter genes (e.g., luciferase or fluorescent protein such as GFP or mCherry), which are expressed upon entering target cells. The amount of PsV entry into cells is theoretically proportional to the expression of the reporter genes, enabling quantitative analysis through imaging, flow cytometry, or luciferase assay [82]. Other reporter systems, such as secreted alkaline phosphatase (SEAP), have also been employed in pseudovirus assays, though luciferase and fluorescent proteins remain the most widely used due to their sensitivity, ease of detection, and compatibility with high-throughput workflows [83]. The choice of reporter can be tailored to laboratory instrumentation, throughput requirements, and desired readout modality, highlighting the versatility of pseudovirus neutralization assays across different research and clinical settings. Common packaging systems include lentivirus, vesicular stomatitis virus (VSV), and murine leukemia virus (MLV) vectors, with the first two being more widely used.

Lentivirus vectors derived from human immunodeficiency virus-1 (HIV-1) only retain essential structural and functional genes, with their genomes split across multiple plasmids to prevent replication and enhance safety [84]. The SARS-CoV-2 lentivirus PsV is designed with the spike protein in the envelope plasmid. A lentiviral pseudovirus neutralization assay developed at Duke University (hereafter referred to as the Duke University assay) employs a full-length SARS-CoV-2 spike and a firefly luciferase reporter in HEK293T cells overexpressing ACE2 [9]. The Monogram Biosciences assay is similar but is performed in HEK293T cells overexpressing both ACE2 and TMPRSS2, which produces higher assay signals [70]. Both assays have been clinically validated and adapted in clinical trials. Alternative reporters include nanoluciferase (NanoLuc), yielding 100-fold brighter readout and facilitating the detection of low-titer samples, and fluorescent genes like GFP or ZsGreen [85,86].

VSV PsVs are generated from the replication-incomplete recombinant VSV with glycoprotein (G) gene deletion (rVSV-ΔG) [87]. This system can bud normally from host cells transfected with a plasmid encoding either VSV G protein or other viral surface proteins, which can determine PsV infectivity [87]. A study developed and validated a VSV-based neutralization assay using a GFP reporter, where a C-terminal truncation of the spike protein improved infection efficiency [68]. This assay correlated well with the live-virus neutralization assay (R^2^ = 0.8396) and showed good reproducibility (intra- and inter-assay CVs of 7.2% and 9.2%) [68]. A similar luciferase-based VSV assay also showed strong reproducibility [69].

The lentiviral assays developed at Duke University by Dr. Montefiori’s team and Monogram have been widely utilized in clinical trials [70]. In the phase 3 trial (NCT04470427), the Duke University assay was used to evaluate mRNA-1273 vaccine-elicited neutralization in adults and children [55]. Meanwhile, the Monogram assay was used for NVX-CoV2373 (NCT04611802) [88]. In addition to these lentiviral assays, a phase-I/II trial in Germany (NCT04380701, EudraCT: 2020-001038-36) included a VSV-based neutralization assay for the BNT162b2 vaccine [89]. Some clinical trials, like the phase I trial for the mRNA-1283 vaccine (NCT04813796), incorporate both pseudovirus neutralization assays and live-virus neutralization assays [90]. These studies demonstrate the utility of these assays, though further optimization and standardization are needed to improve comparability.

#### Optimization of Pseudovirus Neutralization Tests

Several factors, including cell line selection, viral system selection, assay duration, and detection method, should be cautiously considered for assay optimization. Various research groups have identified specific cell lines as particularly sensitive in different assays [68,69]. One study identified that BHK21-hACE2 cells were more sensitive to the SARS-CoV-2 PsV packaged by the VSV system, compared to Vero-E6, BHK21, BHK21-hACE2, and 293T cell lines [68]. Another group reported Huh7 cells exhibiting the highest signal of VsV-based PsV infection among six cell lines [69]. Other work identified that 293T-ACE2 cells, with a higher ACE2 expression level, generated a substantially higher signal than Huh7 cells [91]. Of note, the density of the ACE2 receptor on the cell surface could affect the final readout, which should be monitored if using modified cell lines [86].

The choice of viral system also affects assays sensitivity. Lentivirus exhibits a round-shaped morphology (like SARS-CoV-2), while VSV has a bullet-shaped morphology [92,93]. However, VSV PsV is attractive due to the shorter replication time, enabling shorter incubations [69]. Meanwhile, lentiviral systems typically yield lower production titers than VSV systems, prompting the use of ACE2-overexpressing cells to enhance infection. To further improve the packaging efficiency, researchers introduced cytoplasmic tail truncation in the spike protein [94]. However, there are some concerns that C-terminal truncation could mask the function of the D614G mutation [95]. Assay duration is another critical factor. For instance, the lentiviral system typically requires an incubation period of 48–72 h; optimizing this incubation time can improve the signal-to-noise ratio [96]. Moreover, most pseudovirus neutralization assays are performed in 96-well plates with duplicates, limiting throughput. To further expand the test capacity, a study validated an automated VSV-based neutralization assay in a 384-well format, using a fluorescent readout compatible with immunospot readers [97]. Additionally, the use of fluorescent reporter systems enables further multiplexing of pVNT assays. A quadri-fluorescence (qFluo) pseudovirus platform has been developed to simultaneously assess neutralization against multiple SARS-CoV-2 variants, including spike proteins from BA.5, BQ.1.1, XBB.1.5, and CH.1.1 [71]. However, the above assays utilize single-cycle, replication-defective PsVs, while intracellular virus propagation and exit pathways can also have impacts on viral infection.

To better mimic intracellular virus propagation and exit pathways, researchers developed replication-competent VSV-based SARS-CoV-2 chimeric viruses while still being safe enough to work with in BSL-2 facilities due to VSV’s low pathogenicity [98]. These assays showed a comparable correlation with the live-virus neutralization assay [98]. An alternative approach to mimic live SARS-CoV-2 involves using authentic SARS-CoV-2 virus-like particles (VLPs) without the viral genome [99]. VLP-based neutralization assays demonstrated a robust correlation with the live-virus assay (R = 0.874) and significantly shorter incubation time [99]. Moreover, other researchers have enhanced production efficiency by 100-fold through modifications to the peptide structure, achieving minimal variation and strong correlation to the authentic virus (R = 0.90) [100].

While pseudovirus neutralization assays minimize the requirement for a BSL-3 laboratory setting, inherent differences exist between PsVs and authentic viruses. PsVs lack the complete replication machinery and intracellular trafficking of authentic SARS-CoV-2, potentially lowering sensitivity [86]. In addition, the density and folding of the S protein cannot be simulated on PsV particles. Consequently, validating pseudovirus neutralization assays against authentic virus assays is necessary. Given the cell-based nature of these assays, robust optimization, qualification, and standardization are crucial for consistent and comparable results.

### 3.3. Non-Cell-Based Neutralization Tests

Cell-based neutralization tests are complex, variable, and difficult to standardize for large-scale clinical studies. The surrogate virus neutralization test (sVNT) offers a high-throughput, cell- and virus-free alternative that is rapid, automatable, and amenable to standardized workflows. Most SARS-CoV-2 sVNTs measure competitive inhibition between spike subunits and ACE2 using CLIA or ELISA.

The GenScript cPass SARS-CoV-2 Neutralization Antibody Detection Kit was the first commercial sVNT. Sample NAbs blocked HRP-conjugated RBD protein from binding to ACE2-coated ELISA plates, thereby measuring inhibition activity [10]. RBD showed the best binding characteristics of the spike subunits, S1 showed weaker binding, and the N protein did not exhibit dose-dependent specific binding [10]. The assay showed high sensitivity (98%) and specificity (100%) with strong correlations to live-virus (R^2^ = 0.8591) and pseudovirus assays (R^2^ = 0.8374) [10]. The FDA granted EUA for this and similar assays, including the InBios ELISA (targeting full-length spike) and Diazyme CLIA kit (magnetic bead-based RBD-ACE2 inhibition), with reported sensitivities/specificities ranging from ~94% to 98% and low intra-/inter-assay variability (<5%) [16,17,101].

Other non-EUA-approved virus-free multiplex methods include a multiplex fluorescent bead-based assay using S protein trimers with different mutations [102]. It achieved high sensitivity (96.7%), specificity (100%), and strong correlation with live-virus assays (R^2^ = 0.825), while also distinguishing neutralizing responses to different spike mutations. MSD developed commercial chemiluminescent multiplex assays to assess neutralizing activity against multiple SARS-CoV-2 variants, demonstrating strong correlation with PRNT results [103].

While sVNTs are more scalable and standardized than cell-based assays, they measure competitive binding rather than authentic viral neutralization. Moreover, they can only detect designated antibodies, such as those blocking the RBD, rather than all NAbs. Their correlation with authentic virus assays is generally modest [104]. Consequently, sVNTs are often adapted as supplementary tools in clinical trials.

LFAs have also been adapted for assessing neutralizing antibodies, providing a rapid, point-of-care alternative to traditional cell-based assays. While LFAs are usually qualitative, efforts have been made to establish quantitative tests, mostly by assessing the test line intensity [105]. One study developed the COVID-19 NAb-test^TM^ utilizing the same principle as sVNTs, using colloidal-gold-conjugated-RBD and ACE2-coated test lines to detect NAbs [41]. The group reported 79.1-99.8% sensitivity and strong correlation with MNT (R^2^ = 0.80) [41]. Recent developments have enabled tests that not only detect antibodies but also profile them across multiple viral variants in a single strip. An overlaid LFA design demonstrated simultaneous detection of neutralizing antibodies specific to both the Delta and Omicron variants from one test, with good agreement with conventional neutralization assays [106].

Commercially available neutralizing antibody tests are primarily limited to surrogate virus neutralization assays, with representative examples summarized in Table 2. Live-virus neutralization assays are not marketed as off-the-shelf kits due to biosafety level 3 requirements and specialized laboratory expertise. Pseudovirus-based neutralization assays are available as research reagents, providing pre-made pseudoviruses and reporter cell lines, but they typically require in-lab assembly and are less standardized compared with surrogate assays. Neutralization assay selection should be guided by the intended application: live-virus neutralization assays are generally preferred for mechanistic studies requiring high biological fidelity, pseudovirus assays are commonly used in vaccine immunogenicity studies due to their balance of safety and throughput, and surrogate or binding-based assays are more suitable for large-scale epidemiological or clinical trial studies where high-throughput and accessibility are prioritized. However, substantial inter-laboratory variability remains a key limitation of neutralization assays. Differences in assay design, viral systems, and readouts can lead to inconsistent neutralizing antibody measurements across studies, complicating cross-trial comparisons and the establishment of standardized correlates of protection. Efforts to improve assay standardization, calibration, and harmonization are therefore critical to enhance comparability and enable more reliable interpretation of neutralization data across laboratories. Together, these formats allow functional assessment of neutralizing antibodies across different laboratory settings while balancing safety, throughput, and assay complexity.

## 4. Challenges in the Standardization and Clinical Usage of SARS-CoV-2 Neutralization Assays

The biggest challenge in the clinical application is the inconsistency across methods due to the variability of viral strains, assay conditions, and detection techniques. Reference standards are crucial for assay harmonization. The WHO initiated the calibration of international standard substances for SARS-CoV-2 and recruited 27 labs to establish the first WHO IS in December 2020, assigning 1000 International Unit (IU)/mL to the pooled human plasma [15]. The first WHO IS was depleted in the middle of 2021 due to high demand. In 2022, the second WHO IS was established through collaboration among 41 labs, and the reference panel covers the original virus, Alpha, Beta, Gamma, Delta, and Omicron BA.2 [107]. Another study utilized the first WHO IS to calibrate the Duke University and Monogram lentiviral pseudovirus assays, demonstrating progress in assay harmonization [70].

However, various assays have been used in clinical trials. For example, the Phase II/III COV002 trial used a microneutralization assay for the ChAdOx1 nCoV-19 vaccine [78]. The BNT162b1 vaccine trial (NCT04368728) used a modified virus with mNeonGreen reporter [80]. The mRNA-1273 (NCT04470427) and NVX-CoV2373 (NCT04611802) Phase 3 trials employed the Duke and Monogram lentiviral pseudovirus assays, respectively [55,88]. A Phase I/II trial (NCT04380701) in Germany used a VSV-based assay for BNT162b2 [89], while another Phase 1 trial for mRNA-1283 (NCT04813796) used both pseudovirus and live-virus assays [90]. This methodological variability highlights the need for standardization to ensure reliable cross-trial comparisons. Importantly, inter-laboratory variability in assay platforms, readout systems, and antigen design can substantially affect measured neutralizing antibody titers, limiting direct comparability across vaccine studies and complicating the interpretation of correlates of protection.

Furthermore, the SARS-CoV-2 vaccine has been updated for the more recently emerged variants, and 2025-2026 formulations targeting the Omicron JN.1 lineage, preferentially using the LP.8.1 strain, were advised by the FDA for use beginning in fall 2025 [108]. However, international standards for updated variants are still unavailable, complicating assay validation. The gap in standardization remains a significant challenge for consistent vaccine efficacy assessment and informing public health strategies. Future efforts should focus on strengthening international collaborations and developing harmonized frameworks for assay validation and data reporting, particularly in the context of emerging variants and updated vaccine formulations, where comparison of data and consistent interpretation of immune responses is critical.

## 5. Choosing Between Antibody Binding and Neutralization Assays

Antibody binding assays and neutralization assays are the primary methods to study the immune response to SARS-CoV-2. Singleplex and multiplex binding assays detect and quantify antibodies by measuring their specific binding to target proteins like spike or nucleocapsid. These sensitive methods identify different antibody types (IgM, IgG, IgA), whose kinetics have been extensively studied. Antibody binding assays can be quickly modified to detect variant-specific antibodies. Multiplex assays enable simultaneous measurement of antibodies against multiple viral antigens, variants, and other circulating coronaviruses, maximizing data from a single sample. This provides deeper insights into infection severity and differentiates vaccine-induced responses from infection-induced responses. Antibody binding assays operate safely under BSL-2 conditions, are rapid, and scalable with automation. Numerous antibody binding assays have been validated and described in the literature, and the FDA has authorized several assays under EUAs. Results can be calibrated with the WHO IS and expressed in BAU/mL [109]. Accordingly, these assays are widely used in research studies and clinical settings, particularly for vaccine clinical trials. However, antibody binding assays cannot distinguish between neutralizing and non-neutralizing antibodies, which may affect estimates of immunity.

Importantly, antibody binding assays capture the total humoral response, including non-neutralizing antibodies (nNAbs), which may contribute to antiviral immunity through Fc-mediated effector functions [110]. These non-neutralizing antibodies do not directly block infection but can promote viral clearance via mechanisms such as antibody-dependent cellular cytotoxicity (ADCC), antibody-dependent cellular phagocytosis (ADCP), and complement activation [110]. In addition, non-neutralizing antibodies recognizing more conserved viral epitopes, such as the N protein, can potentially contribute to cross-reactive immunity against emerging variants [111]. Functional activities associated with non-neutralizing antibodies require specialized assays for evaluation, which are outside of the scope of this review.

Neutralization assays measure the ability of antibodies to block viral entry primarily via the ACE2 receptor, reflecting functional activity rather than total binding levels. This category includes live-virus, pseudovirus, and surrogate neutralization assays. Live-virus neutralization assays like PRNT are the gold standard for quantifying functional antibodies, but require BSL-3 facilities, limiting their accessibility. On the other hand, pseudovirus and surrogate assays may be tested at a reduced biosafety level (BSL-2), although they may not fully replicate the behavior of live viruses. Neutralization assays can evaluate variant-specific responses by incorporating corresponding spike mutations, allowing for the assessment of immune escape. However, these assays lack standardization and exhibit high variability across laboratories, complicating cross-study comparisons. Live-virus assays can further contribute to the variability due to manual readouts and differences in viral stocks and cell lines. Calibration of neutralization assays to international standards, as established for antibody binding assays, could help mitigate some of these issues, improving comparability across studies. Additionally, neutralization assays typically have lower throughput and are labor-intensive, although pseudovirus and surrogate assays have improved scalability. Neutralization assays are commonly used in research laboratories, especially for assessing vaccine efficacy, the durability of immune responses, and the cross-neutralization of viral variants.

Antibody binding assays offer convenience and broad clinical utility, while neutralization assays provide detailed functional immunity insights (Table 3). For a highly mutable virus like SARS-CoV-2, neutralization assays are particularly important, as antibodies detected by ELISAs against the original spike protein may cross-react with multiple variants without necessarily neutralizing them. Studies show that binding antibody levels often correlate with neutralization titers, making antibody binding assays useful surrogates when neutralization assays are not feasible due to laboratory capabilities, cost, or low throughput. However, the strength of this correlation varies across studies, depending on factors such as assay format and robustness, antigen selection (e.g., full-length spike versus RBD), population characteristics, and timing of sample collection relative to infection or vaccination [44,112,113]. Differences have also been reported between convalescent and post-vaccination sera, contributing to variability in the relationship between binding and neutralizing antibodies [114]. In addition, viral evolution further contributes to this variability, as emerging variants with substantial antigenic changes can differentially affect binding and neutralizing antibody responses, leading to reduced concordance in some settings [42,115]. Harmonization efforts have focused on aligning serological assays with international standards to improve comparability [109].

Moreover, binding assays may overestimate protective immunity when non-neutralizing antibodies dominate the response, as high binding titers do not necessarily translate into effective viral neutralization. Conversely, neutralization assays may underestimate aspects of protective immunity by failing to capture Fc-mediated functions mediated by nNAbs, particularly when antibody-dependent effector mechanisms contribute to viral clearance. In practice, assay selection should be guided by the study objective: binding assays are typically preferred for high-throughput surveillance, epidemiological studies, and vaccine immunogenicity studies, whereas neutralization assays are often performed in parallel to establish correlation with binding and are regarded as the gold standard for assessing viral neutralization, given their essential role in functional characterization, mechanistic studies, and evaluation of variant-specific immunity. An integrated approach combining binding and neutralization assays, supplemented by functional methods assessing non-neutralizing activities and cellular immune assays when feasible, provides the most comprehensive evaluation of immune responses to SARS-CoV-2.

## 6. Conclusions

Antibody binding assays for SARS-CoV-2 were rapidly developed following publication of the viral antigen sequences. Early assays primarily targeted the spike RBD due to challenges in expressing the full-length spike protein, which were later expanded to include the nucleocapsid and emerging variants. ELISA-based singleplex assays remain cost-effective and accessible for in-house development but are limited by labor-intensive workflows and the need for assay calibration to international standards. Multiplex binding assays maximize information from small sample volumes and reduce analysis time, though higher instrument costs constrain their use. During the pandemic, antibody binding assays acted as early-deployment, rapidly scalable assays, enabling the quick evaluation of immune responses to SARS-CoV-2 and facilitating vaccine development. Neutralization assays complement antibody binding assays by assessing key aspects of functional immunity. While live-virus assays provide the highest biological relevance, their scalability is limited by biosafety requirements. Pseudovirus assays offer safer and faster alternatives using replication-deficient viruses, though they still involve live cells and multiple variables. Surrogate neutralization assays overcome the need for live viruses and cells, providing high-throughput capabilities. However, they assess the competitive binding rather than the functional neutralizing activities. In clinical research studies, the choice depends on the correlations between assays and specific aims and requirements, resources, and testing capacity, with pseudovirus and surrogate assays being increasingly used for large-scale studies and screenings due to their relative practicality.

The integration of binding and neutralization assays, together with complementary approaches, such as functional assays for non-neutralizing antibodies and cellular immune profiling, will be essential to advance our understanding of SARS-CoV-2 immunity and to inform future vaccine strategies. For viruses that undergo continual evolution, such as SARS-CoV-2, ongoing methodological adaptation and the integration of diverse assay platforms are critical to support vaccine evaluation, inform clinical decision-making, and guide public health strategies.

## Figures and Tables

**Table 1:** Antibody binding serology assays for SARS-CoV-2.

Format		Advantages	Limitations	Representative Assays	Antigen(s)	Ig Detection
Singleplex	ELISA	Well established and standardized, high reproducibility	Requires moderate sample volume, single analyte per well	EUROIMMUN Anti-SARS-CoV-2 S1 Curve ELISA (EUA ^a^ originally issued on 4 October 2021, updated on 1 March 2022) [17]	Spike S1(wt)	IgG
				Mount Sinai COVID-19 ELISA IgG Antibody Test (EUA ^a^ originally issued on 15 April 2020) [17]	RBD, Spike (wt)	IgG
				Roy et al., 2020 [14]	RBD (wt SARS-CoV-2, SARS-CoV-1, CoV-HKU1)	IgG, IgM, IgA
				Verkerke et al., 2021 [18]	RBD (wt SARS-CoV-2, NL63, 229E), Spike S1 (HKU1, OC43)	IgG, IgM, IgA
				Abbad et al., 2024 [19]	Spike, Nucleocapsid (wt, BA.1, B.1.1.7, B.1.351, P.1, B.1.617.2, BA.2, BA.5, BQ.1.1, XBB.1)	IgG
				Haynesworth et al., 2024 [11]	Spike, Nucleocapsid (wt)	IgG, IgM
	CLIA	High throughput, sensitive, low background	Sensitivity and specificity vary by antigen, antibody isotype, sample timing, and assay design	Abbott SARS-CoV-2 IgG assay (EUA ^a^ originally issued on 26 April 2020, updated on 24 May 2022) [17]	Spike, Nucleocapsid (wt)	IgG
				Diazyme DZ-Lite SARS-CoV-2 IgM CLIA Kit (EUA ^a^ originally issued on 17 August 2020, updated on 18 January 2022) [17]	Spike, Nucleocapsid (wt)	IgM
				Roche Elecsys Anti-SARS-CoV-2/Anti-SARS-CoV-2 S (EUA ^a^ originally issued on 25 November 2020, updated on 24 August 2022) [17]	RBD, Nucleocapsid (wt)	Pan-Ig
				Beckman Coulter Access anti-SARS-CoV-2 IgG immunoassay [20]	RBD (wt)	IgG
	LFA	Rapid, portable, point-of-care, easy field deployment	Less sensitive, limited quantitation, operator-dependent, lower reproducibility	Access Bio CareStart EZ COVID-19 IgM/IgG (EUA ^a^ originally issued on 24 June 2021, updated on 12 August 2021) [17]	Spike, Nucleocapsid (wt)	IgG, IgM
				Batra et al., 2025 [21]	Influenza A, B and SARS-CoV-2	not specified
Multiplex	Bead-Based	Simultaneous measurement of multiple antigens or Ig isotypes, high dynamic range	Requires specialized instrument, signal saturation, potential bead–bead interference, optimization	Luminex-based xMAP^®^ SARS-CoV-2 Multi-Antigen IgG Assay [22]	S1, RBD, Nucleocapsid (wt)	IgG
				ProcartaPlex panel [23]	Spike trimer, S1, RBD, Nucleocapsid (wt); SARS-CoV-1, MERS, CoV-NL63, CoV-KHU1, CoV-229E, CoV-OC43	Pan-Ig
	Microtitre Plate-Based Arrays	Simultaneous measurement of multiple antigens or Ig isotypes, reduced sample volume	Spot-to-spot variability, crosstalk, requires optimization	MSD panels [24]	Spike, RBD, Nucleocapsid (wt and all variants, including Alpha, Beta, Gamma, Delta, Lambda, Mu, Omicron); SARS-CoV-1, MERS-CoV, circulating Coronaviruses, and other respiratory pathogen	IgG, IgA, IgM

^a^ webpage last updated 28 August 2025. EUA, Emergency Use Authorization. MSD, Meso Scale Discovery. RBD, receptor-binding domain. wt, wild-type, in this case, the Wuhan strain.

**Table 2:** Summary of neutralization assays for SARS-CoV-2.

Format		Advantage	Limitations	Representative Assays	Readout
Live-virus neutralization test	Plaque reduction neutralization test (PRNT)	Gold standard	Low throughput; subjective readout; BSL-3 requirement; biosafety concern	Horbach et al., 2024 [64]	Visual judgment
	Focus reduction neutralization test (FRNT) and microneutralization test (MNT)	Gold standard; accurate readout	Medium throughput; BSL-3 requirement; biosafety concern	Muruato et al., 2020 [65]	Fluorescent signal (mNeonGreen)
				Xie et al., 2020 [66]	Nanoluciferase signal
				Lichtenegger et al., 2022 [67]	qPCR
Pseudovirus neutralization test	VSV-based neutralization test	BSL-2 requirement; shorter incubation time; potential to scale up	Medium throughput; not fully represents the complete replication mechanism; VSV residue may lead to false positive results	Xiong et al., 2020 [68]	Fluorescent signal (GFP)
				Nie et al., 2020 [69]	Luciferase signal
	Lentivirus-based neutralization test	BSL-2 requirement; potential to scale up	Medium throughput; not fully represents the complete replication mechanism; requires ACE2-expressing cells	Duke assay [9]	Luciferase signal
				Monogram assay [70]	Luciferase signal
				Quadri-fluorescence (qFluo) pseudovirus platform [71]	Four fluorescent signals
Cell-free neutralization test	Surrogate virus neutralization test	High throughput; less biosafety concern; cost-efficient;	Evaluates RBD-ACE2 binding, not functional activity	Genscript cPass SARS-CoV-2 Neutralization Antibody Detection Kit (EUA ^a^ originally issued on 6 November 2020, updated on 1 February 2022) [17]	Colormetric signal
				InBios SCoV-2 Detect Neutralizing Ab ELISA (EUA ^a^ originally issued on 22 October 2021) [17]	Colormetric signal
				Diazyme SARS-CoV-2 Neutralizing Antibody CLIA Kit (EUA ^a^ originally issued on 6 December 2022) [17]	Chemiluminescent signal
				MSD multiplex panel [24]	Chemiluminescent signal
	Lateral flow neutralization test	Self-administration; short test time; population screening	Less sensitivity and accuracy; may miss NAbs not targeting the RBD	Fulford et al., 2021 [41]	Visual observation

^a^ webpage last updated 28 August 2025. ACE2, Angiotensin-Converting Enzyme 2. BSL, biosafety level. CLIA, chemiluminescent immunoassay. EUA, Emergency Use Authorization. FRNT, focus reduction neutralization test. GFP, green fluorescent protein. MNT, microneutralization test. MSD, Meso Scale Discovery. NAbs, neutralizing antibodies. PRNT, plaque reduction neutralization test. qPCR, quantitative polymerase chain reaction. RBD, receptor-binding domain. SARS-CoV-2, Severe Acute Respiratory Syndrome Coronavirus 2.

**Table 3:** Comparison between antibody binding assays and neutralization assays.

	Antibody Binding Assays	Neutralization Assays
Detection Target	Antibody binding to viral antigens	Functional inhibition of viral entry
Antigen Targets	Spike protein (S1, RBD), or nucleocapsid (N)	Primarily spike RBD-ACE2 interaction
Assay Formats	ELISA, CLIA, LFA, Multiplex bead-based or microplate arrays	PRNT, pVNT, sVNT
Information Provided	Quantitative or semi-quantitative antibody levels; can differentiate between IgG, IgM, IgA; can resolve IgG subclasses; can capture total humoral response including non-neutralizing antibodies (nNAbs)	Functional assessment of virus-neutralizing capacity, particularly relevant for assessing variant-specific immune escape; indicates potential protective immunity
Turnaround Time	Minutes to hours	Hours to days
Biosafety Level	BSL-2	PRNT: BSL-3; pVNT/sVNT: BSL-2
Throughput	High	Moderate to low
Standardization	Easier to harmonize across labs with national and international standards	More challenging due to the variability of cells and viruses
Limitations	Does not assess functional antibody activity; may cross-react with variants but not indicate neutralization	Biosafety concern, labor-intensive, lower throughput

ACE2, Angiotensin-Converting Enzyme 2. BSL, biosafety level. ELISA, enzyme-linked immunosorbent assay. N, nucleocapsid. PRNT, plaque reduction neutralization test. pVNT, pseudovirus neutralization test. RBD, receptor-binding domain. sVNT, surrogate virus neutralization test.

## Data Availability

Data are contained within the article.

## Abbreviations

The following abbreviations are used in this manuscript:

ACE2	Angiotensin-Converting Enzyme 2
ADCC	Antibody-Dependent Cellular Cytotoxicity
ADCP	Antibody-Dependent Cellular Phagocytosis
ARDS	Acute Respiratory Distress Syndrome
BAU/mL	Binding Antibody Units per milliliter
BSL	Biosafety Level
CLIA	Chemiluminescence Immunoassay
COVID-19	Coronavirus Disease 2019
CPE	CytoPathic Effect
CV	Coefficient of Variation
E	Envelope (protein)
ELISA	Enzyme-Linked Immunosorbent Assay
EUA	Emergency Use Authorization
FDA	U.S. Food and Drug Administration
FRNT	Focus Reduction Neutralization Test
GFP	Green Fluorescent Protein
HCoV	Human Coronavirus
HIV-1	Human Immunodeficiency Virus-1
HRP	Horseradish Peroxidase
Ig	Immunoglobulin
IgA	Immunoglobulin A
IgG	Immunoglobulin G
IgM	Immunoglobulin M
IS	International Standard
IU	International Unit
LFA	Lateral Flow Assay/Immunoassay
M	Membrane (protein)
MERS-CoV	Middle East Respiratory Syndrome Coronavirus
MFI	Median Fluorescence Intensity
MLV	Murine Leukemia Virus
MNT	Microneutralization Test
MSD	Meso Scale Discovery
N	Nucleocapsid (protein)
NAbs	Neutralizing Antibodies
nNAbs	Non-Neutralizing Antibodies
NSCLC	Non-Small-Cell Lung Cancer
NTD	N-Terminal Domain
PRNT	Plaque Reduction Neutralization Test
PsV	Pseudovirus
pVNT	Pseudovirus Neutralization Test
qRT-PCR	Quantitative Reverse Transcription Polymerase Chain Reaction
RBD	Receptor-Binding Domain
RT-PCR	Reverse Transcription Polymerase Chain Reaction
S	Spike (protein)
SARS-CoV-1	Severe Acute Respiratory Syndrome Coronavirus 1
SARS-CoV-2	Severe Acute Respiratory Syndrome Coronavirus 2
SEAP	Secreted Alkaline Phosphatase
SOP	Standard Operating Procedure
sVNT	Surrogate Virus Neutralization Test
TMB	TetraMethylBenzidine
TMPRSS2	Transmembrane Serine Protease 2
VLP	Virus-Like Particle
VSV	Vesicular Stomatitis Virus
WHO	World Health Organization
wt	Wild-type

## References

[1] WHO WHO COVID-19 Dashboard. https://data.who.int/dashboards/covid19/cases.

[2] Dennis A., Wamil M., Alberts J., Oben J., Cuthbertson D.J., Wootton D., Crooks M., Gabbay M., Brady M., Hishmeh L. (2021). Multiorgan impairment in low-risk individuals with post-COVID-19 syndrome: A prospective, community-based study. BMJ Open.

[3] Lamers M.M., Haagmans B.L. (2022). SARS-CoV-2 pathogenesis. Nat. Rev. Microbiol..

[4] Munipalli B., Seim L., Dawson N.L., Knight D., Dabrh A.M.A. (2022). Post-acute sequelae of COVID-19 (PASC): A meta-narrative review of pathophysiology, prevalence, and management. SN Compr. Clin. Med..

[5] Yang H., Rao Z. (2021). Structural biology of SARS-CoV-2 and implications for therapeutic development. Nat. Rev. Microbiol..

[6] Stadlbauer D., Amanat F., Chromikova V., Jiang K., Strohmeier S., Arunkumar G.A., Tan J., Bhavsar D., Capuano C., Kirkpatrick E. (2020). SARS-CoV-2 Seroconversion in Humans: A Detailed Protocol for a Serological Assay, Antigen Production, and Test Setup. Curr. Protoc. Microbiol..

[7] Hober S., Hellstrom C., Olofsson J., Andersson E., Bergstrom S., Jernbom Falk A., Bayati S., Mravinacova S., Sjoberg R., Yousef J. (2021). Systematic evaluation of SARS-CoV-2 antigens enables a highly specific and sensitive multiplex serological COVID-19 assay. Clin. Transl. Immunol..

[8] Shengule S., Alai S., Bhandare S., Patil S., Gautam M., Mangaonkar B., Gupta S., Shaligram U., Gairola S. (2024). Validation and Suitability Assessment of Multiplex Mesoscale Discovery Immunogenicity Assay for Establishing Serological Signatures Using Vaccinated, Non-Vaccinated and Breakthrough SARS-CoV-2 Infected Cases. Vaccines.

[9] Shen X., Tang H., McDanal C., Wagh K., Fischer W., Theiler J., Yoon H., Li D., Haynes B.F., Sanders K.O. (2021). SARS-CoV-2 variant B.1.1.7 is susceptible to neutralizing antibodies elicited by ancestral spike vaccines. Cell Host Microbe.

[10] Tan C.W., Chia W.N., Qin X., Liu P., Chen M.I., Tiu C., Hu Z., Chen V.C., Young B.E., Sia W.R. (2020). A SARS-CoV-2 surrogate virus neutralization test based on antibody-mediated blockage of ACE2-spike protein-protein interaction. Nat. Biotechnol..

[11] Haynesworth K., Kemp T.J., Loftus S., Metz J., Castro N.C., Bullock J., Fetterer D., Pinto L.A. (2024). Analytical measuring interval, linearity, and precision of serology assays for detection of SARS-CoV-2 antibodies according to CLSI guidelines. mSphere.

[12] Amellal H., Assaid N., Charoute H., Akarid K., Maaroufi A., Ezzikouri S., Sarih M. (2023). Kinetics of specific anti-SARS-CoV-2 IgM, IgA, and IgG responses during the first 12 months after SARS-CoV-2 infection: A prospective longitudinal study. PLoS ONE.

[13] Ha B., Jadhao S., Hussaini L., Gibson T., Stephens K., Salazar L., Ciric C., Taylor M., Rouphael N., Edupuganti S. (2021). Evaluation of a SARS-CoV-2 Capture IgM Antibody Assay in Convalescent Sera. Microbiol. Spectr..

[14] Roy V., Fischinger S., Atyeo C., Slein M., Loos C., Balazs A., Luedemann C., Astudillo M.G., Yang D., Wesemann D.R. (2020). SARS-CoV-2-specific ELISA development. J. Immunol. Methods.

[15] NIBSC (2020). WHO International Standard First WHO International Standard for Anti-SARS-CoV-2 Immunoglobulin (Human) NIBSC Code: 20/136 Instructions for Use (Version 2.0, Dated 17/12/2020). https://nibsc.org/documents/ifu/20-136.pdf.

[16] U.S. Food & Drug Administration EUA Authorized Serology Test Performance. https://www.fda.gov/medical-devices/covid-19-emergency-use-authorizations-medical-devices/eua-authorized-serology-test-performance.

[17] U.S. Food & Drug Administration In Vitro Diagnostics Emergency Use Authorizations (EUAs)—Serology and Other Adaptive Immune Response Tests for SARS-CoV-2. https://www.fda.gov/medical-devices/covid-19-emergency-use-authorizations-medical-devices/in-vitro-diagnostics-emergency-use-authorizations-euas-serology-and-other-adaptive-immune-response.

[18] Verkerke H., Horwath M., Saeedi B., Boyer D., Allen J.W., Owens J., Arthur C.M., Nakahara H., Rha J., Patel K. (2021). Comparison of Antibody Class-Specific SARS-CoV-2 Serologies for the Diagnosis of Acute COVID-19. J. Clin. Microbiol..

[19] Abbad A., Yellin T., Singh G., Fried M., Raskin A., Tcheou J., Monahan B., Gleason C., Simon V., Carreño J.M. (2024). SARS-CoV-2 BA.1 and BA.2 breakthrough infections boost antibody responses to early Omicron subvariants but not BQ.1.1 or XBB.1.5. Cell Rep. Med..

[20] Ruscio M., D’Agnolo E., Belgrano A., Plebani M., Lippi G. (2020). Analytical assessment of Beckman Coulter Access anti-SARS-CoV-2 IgG immunoassay. J. Lab. Precis. Med..

[21] Batra R., Blandford E., Kulasegaran-Shylini R., Futschik M.E., Bown A., Catton M., Conti-Frith H., Alexandridou A., Gill R., Milroy C. (2025). Multiplex lateral flow test sensitivity and specificity in detecting influenza A, B and SARS-CoV-2 in adult patients in a UK emergency department. Emerg. Med. J..

[22] Das S., Dunbar S. (2023). Multiplex Immunoassay Approaches Using Luminex(R) xMAP(R) Technology for the Study of COVID-19 Disease. Adv. Exp. Med. Biol..

[23] Thermo Fisher Scientific Coronavirus Serology Assays: Immunoassays for Research. https://www.thermofisher.com/us/en/home/life-science/antibodies/immunoassays/immunoassay-research-areas/coronavirus-serology-assays.html.

[24] MSD Tools for COVID-19 Research. https://www.mesoscale.com/en/products_and_services/assay_kits/covid-19.

[25] Pernet O., Balog S., Kawaguchi E.S., Lam C.N., Anthony P., Simon P., Kotha R., Sood N., Hu H., Kovacs A. (2023). Quantification of Severe Acute Respiratory Syndrome Coronavirus 2 Binding Antibody Levels To Assess Infection and Vaccine-Induced Immunity Using WHO Standards. Microbiol. Spectr..

[26] Cohen K.W., Linderman S.L., Moodie Z., Czartoski J., Lai L., Mantus G., Norwood C., Nyhoff L.E., Edara V.V., Floyd K. (2021). Longitudinal analysis shows durable and broad immune memory after SARS-CoV-2 infection with persisting antibody responses and memory B and T cells. Cell Rep. Med..

[27] Long Q.X., Liu B.Z., Deng H.J., Wu G.C., Deng K., Chen Y.K., Liao P., Qiu J.F., Lin Y., Cai X.F. (2020). Antibody responses to SARS-CoV-2 in patients with COVID-19. Nat. Med..

[28] Burbelo P.D., Riedo F.X., Morishima C., Rawlings S., Smith D., Das S., Strich J.R., Chertow D.S., Davey R.T., Cohen J.I. (2020). Sensitivity in Detection of Antibodies to Nucleocapsid and Spike Proteins of Severe Acute Respiratory Syndrome Coronavirus 2 in Patients With Coronavirus Disease 2019. J. Infect. Dis..

[29] Jarlhelt I., Perez-Alos L., Bayarri-Olmos R., Hansen C.B., Petersen M.S., Weihe P., Armenteros J.J.A., Madsen J.R., Nielsen J.P.S., Hilsted L.M. (2023). Distinguishing SARS-CoV-2 infection and vaccine responses up to 18 months post-infection using nucleocapsid protein and receptor-binding domain antibodies. Microbiol. Spectr..

[30] Cheng S.M.S., Lau J.J., Tsang L.C.H., Leung K., Lee C.K., Hachim A., Kavian N., Chaothai S., Wong R.W.K., Yu J.K.M. (2024). Serological assays for differentiating natural COVID-19 infection from vaccine induced immunity. J. Clin. Virol..

[31] Liu B., Su X., Yu G., Yang S., Wang F., Huang T., Zhou L., Hui Z., Liao Y., Qiu Y. (2022). An automated chemiluminescent immunoassay (CLIA) detects SARS-CoV-2 neutralizing antibody levels in COVID-19 patients and vaccinees. Int. J. Infect. Dis..

[32] Padoan A., Cosma C., Sciacovelli L., Faggian D., Plebani M. (2020). Analytical performances of a chemiluminescence immunoassay for SARS-CoV-2 IgM/IgG and antibody kinetics. Clin. Chem. Lab. Med..

[33] Vilca-Alosilla J.J., Candia-Puma M.A., Coronel-Monje K., Goyzueta-Mamani L.D., Galdino A.S., Machado-de-Avila R.A., Giunchetti R.C., Ferraz Coelho E.A., Chavez-Fumagalli M.A. (2023). A Systematic Review and Meta-Analysis Comparing the Diagnostic Accuracy Tests of COVID-19. Diagnostics.

[34] Zheng X., Duan R.H., Gong F., Wei X., Dong Y., Chen R., Yue Liang M., Tang C., Lu L. (2022). Accuracy of serological tests for COVID-19: A systematic review and meta-analysis. Front. Public Health.

[35] Speletas M., Kyritsi M.A., Vontas A., Theodoridou A., Chrysanthidis T., Hatzianastasiou S., Petinaki E., Hadjichristodoulou C. (2020). Evaluation of Two Chemiluminescent and Three ELISA Immunoassays for the Detection of SARS-CoV-2 IgG Antibodies: Implications for Disease Diagnosis and Patients’ Management. Front. Immunol..

[36] Coste A.T., Jaton K., Papadimitriou-Olivgeris M., Greub G., Croxatto A. (2021). Comparison of SARS-CoV-2 serological tests with different antigen targets. J. Clin. Virol..

[37] Lokida D., Karyana M., Kosasih H., Mardian Y., Sugiyono R.I., Arlinda D., Lukman N., Salim G., Butar Butar D.P., Naysilla A.M. (2022). Performance and correlation of ten commercial immunoassays for the detection of SARS-CoV-2 antibodies. Heliyon.

[38] Ju J., Zhang X., Li L., Regmi S., Yang G., Tang S. (2022). Development of fluorescent lateral flow immunoassay for SARS-CoV-2-specific IgM and IgG based on aggregation-induced emission carbon dots. Front. Bioeng. Biotechnol..

[39] Mlcochova P., Collier D., Ritchie A., Assennato S.M., Hosmillo M., Goel N., Meng B., Chatterjee K., Mendoza V., Temperton N. (2020). Combined Point-of-Care Nucleic Acid and Antibody Testing for SARS-CoV-2 following Emergence of D614G Spike Variant. Cell Rep. Med..

[40] Vengesai A., Midzi H., Kasambala M., Mutandadzi H., Mduluza-Jokonya T.L., Rusakaniko S., Mutapi F., Naicker T., Mduluza T. (2021). A systematic and meta-analysis review on the diagnostic accuracy of antibodies in the serological diagnosis of COVID-19. Syst. Rev..

[41] Fulford T.S., Van H., Gherardin N.A., Zheng S., Ciula M., Drummer H.E., Redmond S., Tan H.X., Boo I., Center R.J. (2021). A point-of-care lateral flow assay for neutralising antibodies against SARS-CoV-2. EBioMedicine.

[42] Higashimoto Y., Kozawa K., Miura H., Kawamura Y., Ihira M., Hiramatsu H., Suzuki R., Haga K., Takai-Todaka R., Sawada A. (2022). Correlation between anti-S IgG and neutralizing antibody titers against three live SARS-CoV-2 variants in BNT162b2 vaccine recipients. Hum. Vaccin. Immunother..

[43] Takheaw N., Liwsrisakun C., Chaiwong W., Laopajon W., Pata S., Inchai J., Duangjit P., Pothirat C., Bumroongkit C., Deesomchok A. (2022). Correlation Analysis of Anti-SARS-CoV-2 RBD IgG and Neutralizing Antibody against SARS-CoV-2 Omicron Variants after Vaccination. Diagnostics.

[44] Souiri A., Lemriss S., El Maliki B., Falahi H., El Fahime E., El Kabbaj S. (2023). SARS-CoV-2-Neutralizing Antibody Response and Correlation of Two Serological Assays with Microneutralization. Vaccines.

[45] Schest S., Langer C., Stiegler Y., Karnuth B., Arends J., Stiegler H., Masetto T., Peter C., Grimmler M. (2023). Vaccine-induced SARS-CoV-2 antibody response: The comparability of S1-specific binding assays depends on epitope and isotype discrimination. Front. Immunol..

[46] Honning A., Tomczyk S., Hermes J., Grossegesse M., Hofmann N., Michel J., Neumann M., Nitsche A., Hoppe B., Eckmanns T. (2024). Follow-up SARS-CoV-2 serological study of a health care worker cohort following COVID-19 booster vaccination. BMC Infect. Dis..

[47] Angeloni S., Cordes R., Dunbar S., Garcia C., Gibson G., Martin C., Stone V. (2013). xMAP^®^ Cookbook. A Collection of Methods and Protocols for Developing Multiplex Assays with xMAP Technology.

[48] Diasorin (2024). xMAP^®^ INTELLIFLEX Catalog. https://int.diasorin.com/sites/default/files/products-documentation-tool/BR663101.xMAPCatalog.2024.EMEA_.1223WR.pdf.

[49] Rottmayer K., Schwarze M., Jassoy C., Hoffmann R., Loeffler-Wirth H., Lehmann C. (2024). Potential of a Bead-Based Multiplex Assay for SARS-CoV-2 Antibody Detection. Biology.

[50] Roy D.R., Kemp T.J., Haynesworth K., Loftus S.A., Pinto L.A. (2023). Development, Validation, and Utilization of a Luminex-Based SARS-CoV-2 Multiplex Serology Assay. Microbiol. Spectr..

[51] MSD (2025). V-PLEX COVID-19 Serology Kits. https://www.mesoscale.com/~/media/files/product%20inserts/v-plex%20covid-19%20serology%20assays%20insert.pdf.

[52] Byrum J.R., Waltari E., Janson O., Guo S.M., Folkesson J., Chhun B.B., Vinden J., Ivanov I.E., Forst M.L., Li H. (2023). MultiSero: An Open-Source Multiplex-ELISA Platform for Measuring Antibody Responses to Infection. Pathogens.

[53] Tighe P.J., Ryder R.R., Todd I., Fairclough L.C. (2015). ELISA in the multiplex era: Potentials and pitfalls. Proteom. Clin. Appl..

[54] Falsey A.R., Sobieszczyk M.E., Hirsch I., Sproule S., Robb M.L., Corey L., Neuzil K.M., Hahn W., Hunt J., Mulligan M.J. (2021). Phase 3 Safety and Efficacy of AZD1222 (ChAdOx1 nCoV-19) COVID-19 Vaccine. N. Engl. J. Med..

[55] Gilbert P.B., Montefiori D.C., McDermott A.B., Fong Y., Benkeser D., Deng W., Zhou H., Houchens C.R., Martins K., Jayashankar L. (2022). Immune correlates analysis of the mRNA-1273 COVID-19 vaccine efficacy clinical trial. Science.

[56] Majdoubi A., Michalski C., O’Connell S.E., Dada S., Narpala S., Gelinas J., Mehta D., Cheung C., Winkler D.F., Basappa M. (2021). A majority of uninfected adults show preexisting antibody reactivity against SARS-CoV-2. JCI Insight.

[57] Haddad N.S., Nguyen D.C., Kuruvilla M.E., Morrison-Porter A., Anam F., Cashman K.S., Ramonell R.P., Kyu S., Saini A.S., Cabrera-Mora M. (2021). One-Stop Serum Assay Identifies COVID-19 Disease Severity and Vaccination Responses. Immunohorizons.

[58] Irrgang P., Gerling J., Kocher K., Lapuente D., Steininger P., Habenicht K., Wytopil M., Beileke S., Schafer S., Zhong J. (2023). Class switch toward noninflammatory, spike-specific IgG4 antibodies after repeated SARS-CoV-2 mRNA vaccination. Sci. Immunol..

[59] Martin Perez C., Ruiz-Rius S., Ramirez-Morros A., Vidal M., Opi D.H., Santamaria P., Blanco J., Vidal-Alaball J., Beeson J.G., Molinos-Albert L.M. (2025). Post-vaccination IgG4 and IgG2 class switch associates with increased risk of SARS-CoV-2 infections. J. Infect..

[60] Aurelia L.C., Purcell R.A., Theisen R.M., Kelly A., Esterbauer R., Ramanathan P., Lee W.S., Wines B.D., Hogarth P.M., Juno J.A. (2025). Increased SARS-CoV-2 IgG4 has variable consequences dependent upon Fc function, Fc receptor polymorphism, and viral variant. Sci. Adv..

[61] Yates J.L., Ehrbar D.J., Hunt D.T., Girardin R.C., Dupuis A.P., Payne A.F., Sowizral M., Varney S., Kulas K.E., Demarest V.L. (2021). Serological analysis reveals an imbalanced IgG subclass composition associated with COVID-19 disease severity. Cell Rep. Med..

[62] Siebner A.S., Griesbaum J., Huus K.E., Flugge J., Hopfensperger K., Michel T., Schneiderhan-Marra N., Sauter D., Kremsner P.G., Ley R.E. (2025). Class switch toward IgG2 and IgG4 is more pronounced in BNT162b2 compared to mRNA-1273 COVID-19 vaccinees. Int. J. Infect. Dis..

[63] Valanparambil R.M., Carlisle J., Linderman S.L., Akthar A., Millett R.L., Lai L., Chang A., McCook-Veal A.A., Switchenko J., Nasti T.H. (2022). Antibody Response to COVID-19 mRNA Vaccine in Patients With Lung Cancer After Primary Immunization and Booster: Reactivity to the SARS-CoV-2 WT Virus and Omicron Variant. J. Clin. Oncol..

[64] Horbach I.S., de Souza Azevedo A., Schwarcz W.D., Alves N.D.S., de Moura Dias B., Setatino B.P., da Cruz Moura L., de Souza A.F., Denani C.B., da Silva S.A. (2024). Plaque Reduction Neutralization Test (PRNT) Accuracy in Evaluating Humoral Immune Response to SARS-CoV-2. Diseases.

[65] Muruato A.E., Fontes-Garfias C.R., Ren P., Garcia-Blanco M.A., Menachery V.D., Xie X., Shi P.Y. (2020). A high-throughput neutralizing antibody assay for COVID-19 diagnosis and vaccine evaluation. Nat. Commun..

[66] Xie X., Muruato A.E., Zhang X., Lokugamage K.G., Fontes-Garfias C.R., Zou J., Liu J., Ren P., Balakrishnan M., Cihlar T. (2020). A nanoluciferase SARS-CoV-2 for rapid neutralization testing and screening of anti-infective drugs for COVID-19. Nat. Commun..

[67] Lichtenegger S., Saiger S., Hardt M., Kulnik S., Wagner G.E., Kleinhappl B., Assig K., Zauner A., Ober M., Kimpel J. (2022). Development of a Rapid Live SARS-CoV-2 Neutralization Assay Based on a qPCR Readout. J. Clin. Microbiol..

[68] Xiong H.L., Wu Y.T., Cao J.L., Yang R., Liu Y.X., Ma J., Qiao X.Y., Yao X.Y., Zhang B.H., Zhang Y.L. (2020). Robust neutralization assay based on SARS-CoV-2 S-protein-bearing vesicular stomatitis virus (VSV) pseudovirus and ACE2-overexpressing BHK21 cells. Emerg. Microbes Infect..

[69] Nie J., Li Q., Wu J., Zhao C., Hao H., Liu H., Zhang L., Nie L., Qin H., Wang M. (2020). Establishment and validation of a pseudovirus neutralization assay for SARS-CoV-2. Emerg. Microbes Infect..

[70] Huang Y., Borisov O., Kee J.J., Carpp L.N., Wrin T., Cai S., Sarzotti-Kelsoe M., McDanal C., Eaton A., Pajon R. (2021). Calibration of two validated SARS-CoV-2 pseudovirus neutralization assays for COVID-19 vaccine evaluation. Sci. Rep..

[71] Chen J., Huang Z., Xiao J., Du S., Bu Q., Guo H., Ye J., Chen S., Gao J., Li Z. (2024). A quadri-fluorescence SARS-CoV-2 pseudovirus system for efficient antigenic characterization of multiple circulating variants. Cell Rep. Methods.

[72] Okba N.M.A., Müller M.A., Li W., Wang C., GeurtsvanKessel C.H., Corman V.M., Lamers M.M., Sikkema R.S., de Bruin E., Chandler F.D. (2020). Severe Acute Respiratory Syndrome Coronavirus 2-Specific Antibody Responses in Coronavirus Disease Patients. Emerg. Infect. Dis..

[73] Amanat F., White K.M., Miorin L., Strohmeier S., McMahon M., Meade P., Liu W.C., Albrecht R.A., Simon V., Martinez-Sobrido L. (2020). An In Vitro Microneutralization Assay for SARS-CoV-2 Serology and Drug Screening. Curr. Protoc. Microbiol..

[74] Liu K.T., Han Y.J., Wu G.H., Huang K.A., Huang P.N. (2022). Overview of Neutralization Assays and International Standard for Detecting SARS-CoV-2 Neutralizing Antibody. Viruses.

[75] Vanderheiden A., Edara V.V., Floyd K., Kauffman R.C., Mantus G., Anderson E., Rouphael N., Edupuganti S., Shi P.Y., Menachery V.D. (2020). Development of a Rapid Focus Reduction Neutralization Test Assay for Measuring SARS-CoV-2 Neutralizing Antibodies. Curr. Protoc. Immunol..

[76] Bewley K.R., Coombes N.S., Gagnon L., McInroy L., Baker N., Shaik I., St-Jean J.R., St-Amant N., Buttigieg K.R., Humphries H.E. (2021). Quantification of SARS-CoV-2 neutralizing antibody by wild-type plaque reduction neutralization, microneutralization and pseudotyped virus neutralization assays. Nat. Protoc..

[77] Myler H., Pedras-Vasconcelos J., Lester T., Civoli F., Xu W., Wu B., Vainshtein I., Luo L., Hassanein M., Liu S. (2023). Neutralizing Antibody Validation Testing and Reporting Harmonization. AAPS J..

[78] Ramasamy M.N., Minassian A.M., Ewer K.J., Flaxman A.L., Folegatti P.M., Owens D.R., Voysey M., Aley P.K., Angus B., Babbage G. (2021). Safety and immunogenicity of ChAdOx1 nCoV-19 vaccine administered in a prime-boost regimen in young and old adults (COV002): A single-blind, randomised, controlled, phase 2/3 trial. Lancet.

[79] Kurhade C., Zou J., Xia H., Cai H., Yang Q., Cutler M., Cooper D., Muik A., Jansen K.U., Xie X. (2022). Neutralization of Omicron BA.1, BA.2, and BA.3 SARS-CoV-2 by 3 doses of BNT162b2 vaccine. Nat. Commun..

[80] Mulligan M.J., Lyke K.E., Kitchin N., Absalon J., Gurtman A., Lockhart S., Neuzil K., Raabe V., Bailey R., Swanson K.A. (2020). Phase I/II study of COVID-19 RNA vaccine BNT162b1 in adults. Nature.

[81] Frische A., Brooks P.T., Gybel-Brask M., Sækmose S.G., Jensen B.A., Mikkelsen S., Bruun M.T., Boding L., Strandh C.P., Jørgensen C.S. (2022). Optimization and evaluation of a live virus SARS-CoV-2 neutralization assay. PLoS ONE.

[82] Thimmiraju S.R., Kimata J.T., Pollet J. (2024). Pseudoviruses, a safer toolbox for vaccine development against enveloped viruses. Expert. Rev. Vaccines.

[83] Li Q., Liu Q., Huang W., Li X., Wang Y. (2018). Current status on the development of pseudoviruses for enveloped viruses. Rev. Med. Virol..

[84] Dull T., Zufferey R., Kelly M., Mandel R.J., Nguyen M., Trono D., Naldini L. (1998). A third-generation lentivirus vector with a conditional packaging system. J. Virol..

[85] Crawford K.H.D., Eguia R., Dingens A.S., Loes A.N., Malone K.D., Wolf C.R., Chu H.Y., Tortorici M.A., Veesler D., Murphy M. (2020). Protocol and Reagents for Pseudotyping Lentiviral Particles with SARS-CoV-2 Spike Protein for Neutralization Assays. Viruses.

[86] Schmidt F., Weisblum Y., Muecksch F., Hoffmann H.H., Michailidis E., Lorenzi J.C.C., Mendoza P., Rutkowska M., Bednarski E., Gaebler C. (2020). Measuring SARS-CoV-2 neutralizing antibody activity using pseudotyped and chimeric viruses. J. Exp. Med..

[87] Whitt M.A. (2010). Generation of VSV pseudotypes using recombinant ΔG-VSV for studies on virus entry, identification of entry inhibitors, and immune responses to vaccines. J. Virol. Methods.

[88] Fong Y., Huang Y., Benkeser D., Carpp L.N., Áñez G., Woo W., McGarry A., Dunkle L.M., Cho I., Houchens C.R. (2023). Immune correlates analysis of the PREVENT-19 COVID-19 vaccine efficacy clinical trial. Nat. Commun..

[89] Sahin U., Muik A., Vogler I., Derhovanessian E., Kranz L.M., Vormehr M., Quandt J., Bidmon N., Ulges A., Baum A. (2021). BNT162b2 vaccine induces neutralizing antibodies and poly-specific T cells in humans. Nature.

[90] Yassini P., Hutchens M., Paila Y.D., Schoch L., Aunins A., Siangphoe U., Paris R. (2023). Interim analysis of a phase 1 randomized clinical trial on the safety and immunogenicity of the mRNA-1283 SARS-CoV-2 vaccine in adults. Hum. Vaccin. Immunother..

[91] Neerukonda S.N., Vassell R., Herrup R., Liu S., Wang T., Takeda K., Yang Y., Lin T.L., Wang W., Weiss C.D. (2021). Establishment of a well-characterized SARS-CoV-2 lentiviral pseudovirus neutralization assay using 293T cells with stable expression of ACE2 and TMPRSS2. PLoS ONE.

[92] Boukour S., Massé J.M., Bénit L., Dubart-Kupperschmitt A., Cramer E.M. (2006). Lentivirus degradation and DC-SIGN expression by human platelets and megakaryocytes. J. Thromb. Haemost..

[93] Ge P., Tsao J., Schein S., Green T.J., Luo M., Zhou Z.H. (2010). Cryo-EM model of the bullet-shaped vesicular stomatitis virus. Science.

[94] Johnson M.C., Lyddon T.D., Suarez R., Salcedo B., LePique M., Graham M., Ricana C., Robinson C., Ritter D.G. (2020). Optimized Pseudotyping Conditions for the SARS-COV-2 Spike Glycoprotein. J. Virol..

[95] Chen H.Y., Huang C., Tian L., Huang X., Zhang C., Llewellyn G.N., Rogers G.L., Andresen K., O’Gorman M.R.G., Chen Y.W. (2021). Cytoplasmic Tail Truncation of SARS-CoV-2 Spike Protein Enhances Titer of Pseudotyped Vectors but Masks the Effect of the D614G Mutation. J. Virol..

[96] Cai Z., Kalkeri R., Zhu M., Cloney-Clark S., Haner B., Wang M., Osman B., Dent D., Feng S.L., Longacre Z. (2024). A Pseudovirus-Based Neutralization Assay for SARS-CoV-2 Variants: A Rapid, Cost-Effective, BSL-2-Based High-Throughput Assay Useful for Vaccine Immunogenicity Evaluation. Microorganisms.

[97] Liang Z., Wu X., Wu J., Liu S., Tong J., Li T., Yu Y., Zhang L., Zhao C., Lu Q. (2023). Development of an automated, high-throughput SARS-CoV-2 neutralization assay based on a pseudotyped virus using a vesicular stomatitis virus (VSV) vector. Emerg. Microbes Infect..

[98] Case J.B., Rothlauf P.W., Chen R.E., Liu Z., Zhao H., Kim A.S., Bloyet L.M., Zeng Q., Tahan S., Droit L. (2020). Neutralizing Antibody and Soluble ACE2 Inhibition of a Replication-Competent VSV-SARS-CoV-2 and a Clinical Isolate of SARS-CoV-2. Cell Host Microbe.

[99] Roessler J., Pich D., Albanese M., Wratil P.R., Krähling V., Hellmuth J.C., Scherer C., von Bergwelt-Baildon M., Becker S., Keppler O.T. (2022). Quantitation of SARS-CoV-2 neutralizing antibodies with a virus-free, authentic test. Proc. Natl. Acad. Sci. USA.

[100] Liu S., Zhang L., Fu W., Liang Z., Yu Y., Li T., Tong J., Liu F., Nie J., Lu Q. (2024). Optimization and validation of a virus-like particle pseudotyped virus neutralization assay for SARS-CoV-2. MedComm.

[101] Diazyme Laboratories (2022). Diazyme SARS-CoV-2 Neutralizing Antibody CLIA Kit Instruction For Use.

[102] Fenwick C., Turelli P., Pellaton C., Farina A., Campos J., Raclot C., Pojer F., Cagno V., Nusslé S.G., D’Acremont V. (2021). A high-throughput cell- and virus-free assay shows reduced neutralization of SARS-CoV-2 variants by COVID-19 convalescent plasma. Sci. Transl. Med..

[103] Patil R., Palkar S., Mishra A., Patil R., Arankalle V. (2023). Variable neutralizing antibody responses to 10 SARS-CoV-2 variants in natural infection with wild-type (B.1) virus, Kappa (B.1.617.1), and Delta (B.1.617.2) variants and COVISHIELD vaccine immunization in India: Utility of the MSD platform. Front. Immunol..

[104] Sholukh A.M., Fiore-Gartland A., Ford E.S., Hou Y., Tse L.V., Lempp F.A., Kaiser H., Saint Germain R., Bossard E., Kee J.J. (2020). Evaluation of SARS-CoV-2 neutralization assays for antibody monitoring in natural infection and vaccine trials. medRxiv.

[105] Liu Z., Liang J., Hu H., Wu M., Ma J., Ma Z., Ji J., Chen H., Li X., Wang Z. (2023). Development of an Effective Neutralizing Antibody Assay for SARS-CoV-2 Diagnosis. Int. J. Nanomed..

[106] Deenin W., Khongchareonporn N., Ruxrungtham K., Ketloy C., Hirankarn N., Wangkanont K., Rengpipat S., Yakoh A., Chaiyo S. (2024). Overlaid Lateral Flow Immunoassay for the Simultaneous Detection of Two Variant-Specific SARS-CoV-2 Neutralizing Antibodies. Anal. Chem..

[107] Bentley E. (2022). Establishment of the 2nd WHO International Standard for Anti-SARS-CoV-2 Immunoglobulin and Reference Panel for Antibodies to SARS-CoV-2 Variants of Concern.

[108] U.S. Food & Drug Administration COVID-19 Vaccines (2025–2026 Formula) for Use in the United States Beginning in Fall 2025. https://www.fda.gov/vaccines-blood-biologics/industry-biologics/covid-19-vaccines-2025-2026-formula-use-united-states-beginning-fall-2025.

[109] Kemp T.J., Quesinberry J.T., Cherry J., Lowy D.R., Pinto L.A., Miller M.B. (2022). Selection, Characterization, Calibration, and Distribution of the U.S. Serology Standard for Anti-SARS-CoV-2 Antibody Detection. J. Clin. Microbiol..

[110] Izadi A., Nordenfelt P. (2024). Protective non-neutralizing SARS-CoV-2 monoclonal antibodies. Trends Immunol..

[111] Osuagwu A.E., Payne M., Bosch J., Mbonye U., Nguyen K., Karn J., Bruchez A., King C.L. (2025). Non-neutralizing antibodies against SARS-CoV-2 nucleocapsid protein mediate variant transcendent antibody-dependent cellular cytotoxicity. J. Immunol..

[112] Grunau B., Prusinkiewicz M., Asamoah-Boaheng M., Golding L., Lavoie P.M., Petric M., Levett P.N., Haig S., Barakauskas V., Karim M.E. (2022). Correlation of SARS-CoV-2 Viral Neutralizing Antibody Titers with Anti-Spike Antibodies and ACE-2 Inhibition among Vaccinated Individuals. Microbiol. Spectr..

[113] Cook N., Xu L., Hegazy S., Wheeler B.J., Anderson A.R., Critelli N., Yost M., McElroy A.K., Shurin M.R., Wheeler S.E. (2021). Multiplex assessment of SARS-CoV-2 antibodies improves assay sensitivity and correlation with neutralizing antibodies. Clin. Biochem..

[114] Lerdsamran H., Anusorntanawat R., Sangsiriwut K., Sawadpongpan S., Prasertsopon J., Thinpan N., Intalapaporn P., Techasuwanna R., Okada P., Puthavathana P. (2024). Higher correlation between neutralizing antibodies and surrogate neutralizing or binding antibodies in COVID-19 patients than vaccine recipients. PLoS ONE.

[115] Fu J., Shen X., Anderson M., Stec M., Petratos T., Cloherty G., Montefiori D.C., Landay A., Moy J.N. (2022). Correlation of Binding and Neutralizing Antibodies against SARS-CoV-2 Omicron Variant in Infection-Naive and Convalescent BNT162b2 Recipients. Vaccines.

